# A High-Efficiency Artificial Synthetic Pathway for 5-Aminovalerate Production From Biobased L-Lysine in *Escherichia coli*

**DOI:** 10.3389/fbioe.2021.633028

**Published:** 2021-02-09

**Authors:** Jie Cheng, Wenying Tu, Zhou Luo, Xinghua Gou, Qiang Li, Dan Wang, Jingwen Zhou

**Affiliations:** ^1^Key Laboratory of Meat Processing of Sichuan Province, Key Laboratory of Coarse Cereal Processing, Ministry of Agriculture and Rural Affairs, College of Food and Biological Engineering, Chengdu University, Chengdu, China; ^2^Department of Chemical Engineering, School of Chemistry and Chemical Engineering, Chongqing University, Chongqing, China; ^3^National Engineering Laboratory for Cereal Fermentation Technology, Jiangnan University, Wuxi, China

**Keywords:** 5-aminovalerate, L-Lysine HCl, artificial pathway, molecular dynamic simulation, molecular docking

## Abstract

Bioproduction of 5-aminovalerate (5AVA) from renewable feedstock can support a sustainable biorefinery process to produce bioplastics, such as nylon 5 and nylon 56. In order to achieve the biobased production of 5AVA, a 2-keto-6-aminocaproate-mediated synthetic pathway was established. Combination of L-Lysine α-oxidase from *Scomber japonicus*, α-ketoacid decarboxylase from *Lactococcus lactis* and aldehyde dehydrogenase from *Escherichia coli* could achieve the biosynthesis of 5AVA from biobased L-Lysine in *E. coli*. The H_2_O_2_ produced by L-Lysine α-oxidase was decomposed by the expression of catalase KatE. Finally, 52.24 g/L of 5AVA were obtained through fed-batch biotransformation. Moreover, homology modeling, molecular docking and molecular dynamic simulation analyses were used to identify mutation sites and propose a possible trait-improvement strategy: the expanded catalytic channel of mutant and more hydrogen bonds formed might be beneficial for the substrates stretch. In summary, we have developed a promising artificial pathway for efficient 5AVA synthesis.

## Introduction

Increasing concerns over global water pollution, climate change, public health, and petroleum shortages have attracted considerable attention to sustainable development as promising green alternatives to traditional petrochemical-derived chemicals renewable feedstock (Tsuge et al., 2016). Recently a variety of valuable chemicals such as 6-aminocaproate (Cheng et al., 2019), fructose (Yang et al., 2016), mandelic acid (Youn et al., 2020), vitamin B_12_ (Fang et al., 2018), naringenin (Gao et al., 2020b), *p*-coumaric acid (Gao et al., 2020a), breviscapine (Liu et al., 2018), 4-hydroxybenzoic acid (Klenk et al., 2020), curcuminoids (Rodrigues et al., 2020) and hydroxytyrosol (Zeng et al., 2020) have been produced in microorganisms. As a kind of green alternative to petrochemical products, microbial bioplastics are composed of monomers containing appropriate functional groups, which have become the focus of metabolic engineering research. These compounds include amino acids such as methionine (Kromer et al., 2006) and leucine (Zhang et al., 2008), organic acids such as adipic acid (Zhao et al., 2018a) and glutarate (Zhao et al., 2018b), diamines such as 1,3-diaminopropane (Chae et al., 2015) and diaminopentane (Kind et al., 2010; Rui et al., 2020), as well as diols like 1,3-propanediol (Nakamura and Whited, 2003) and 1,2-propanediol (Niimi et al., 2011). It is worth mentioning that two straight-chain amino acids—5-aminovalerate (5AVA) and 4-aminobutyrate—are promising platform compounds for the synthesis of polyimides, serving as raw materials for disposable goods, clothes and automobile parts like nylon 5 (Adkins et al., 2013) and nylon 4 (Park et al., 2013a) because of its high temperature and organic solvent resistance.

Due to the high demand in the animal feed industry, the production of L-Lysine (L-lys) is saturated today and may even be in oversupply (Vassilev et al., 2018). As one of the most important bulk chemicals, 5AVA has become the precursor for the synthesis of δ-valerolactam (Zhang et al., 2017), glutarate (Rohles et al., 2016; Hong et al., 2018), nylon 5 (Adkins et al., 2013), 5-hydroxyvalerate (Liu et al., 2014) and 1,5-pentanediol (Park et al., 2014). 5AVA is currently produced from petroleum feedstocks with aerobic oxidation of piperidine catalyzed by ceria-supported nanogold (Dairo et al., 2016). However, this chemical synthesis method not only requires higher temperature, but results in greater pollution (Dairo et al., 2016), so it is necessary to discover alternative approaches to produce 5AVA. Recently, with the rapid development of biotechnology, the synthesis of 5AVA by means of metabolic engineering and synthetic biology has attracted more and more attention (Hong et al., 2018).

**FIGURE 1:**
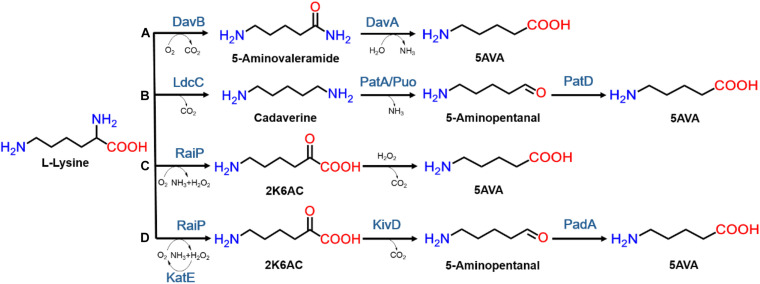
The biosynthesis routes of 5AVA from L-Lysine in microorganisms. The enzymes included in those routes are: **(A)** lysine 2-monooxygenase (DavB), δ-aminovaleramidase (DavA); **(B)** L-Lysine decarboxylase (LdcC), putrescine transaminase (PatA), monooxygenase putrescine oxidase (Puo), and γ-aminobutyraldehyde dehydrogenase (PatD); **(C)** L-Lysine α-oxidase (RaiP); **(D)** L-Lysine α-oxidase (RaiP), α-ketoacid decarboxylase (KivD), catalyze (KatE), and aldehyde dehydrogenase (PadA).

In nature, 5AVA synthesis is closely related to L-lys catabolism in *Pseudomonas putida* (Ying et al., 2017). As seen in Figure 1A, 5AVA was produced through the overexpression of L-lys 2-monooxygenase (DavB) and 5AVA amidohydrolase (DavA) (Joo et al., 2017). According to Park’s report (Park et al., 2013b), 3.6 g/L of 5AVA was succsessfully produced in WL3110/DavA-DavB, but the titer was relatively low. 33.1 g/L of 5AVA was produced under a novel artificial H_36_ promoter in *Corynebacterium glutamicum* (Shin et al., 2016). Interestingly, L-lys specific permease (LysP) has been shown to increase 5AVA titer to 63.2 g/L (Table 1; Li et al., 2016). As seen from Figure 1B, 5AVA has been successfully produced from L-lys via cadaverine-mediated and 5-aminopentanal-mediated pathway (Jorge et al., 2017). With the expression of L-lys α-oxidase (RaiP) from *Scomber japonicus* (*S. japonicus*), 29.12 g/L of 5AVA could be successfully formed from L-lys hydrochloride (L-lys HCl) via 2-keto-6-aminocaproate (2K6AC) as intermediate as seen in Figure 1C (Cheng et al., 2018b). However, the addition of ethanol and H_2_O_2_ were unsafe and uneconomical (Cheng et al., 2018b). 13.4 g/L 5AVA could be successfully obtained with RaiP immobilized on a solid support (Pukin et al., 2010). In addition, 5AVA could be effectively separated by macroporous adsorption resin AK-1 from bioconversion liquid with the purity of 99.3% (Xu et al., 2019).

**TABLE 1:** The production of 5-AVA in different synthetic pathway.

Synthetic pathway	Host strain	Strategy	Description	5AVA titer (g/L)	Yield (g/g)	Substrate/feedstock	References
A	*E. coli*	Whole-cell biotransformation	Expression of DavB and DavA in *E. coli*	240.70	0.70	L-Lysine	Wang et al., 2016
A	*E. coli*	Enzymatic catalysis	Overexpression of DavB, DavA, PP2911 from *P. putida* and LysP from *E. coli*	63.20	0.62	L-Lysine	Li et al., 2016
A	*C. glutamicum*	Fed-batch fermentation	Expression of codon-optimized *davA* and *davB*, promoter engineering	33.10	0.10	Glucose	Shin et al., 2016
A	*C. glutamicum*	Fed-batch fermentation	Pretreatment, hydrolysis, purification and concentration of the *Miscanthus* hydrolyzate solution	12.51	0.10	*Miscanthus* hydrolyzate	Joo et al., 2017
B	*C. glutamicum*	Fermentation	N-acetylcadaverine and glutarate in a genome-streamlined L-Lysine producing strain expressing ldcC, patA, and patD from *E. coli*	5.10	0.13	Glucose and alternative carbon sources	Jorge et al., 2017
B	*C. glutamicum*	Fermentation	*C. glutamicum* GSLA2ΔgabTDP with overexpression of LdcC, Puo, and PatD	3.70	0.09	Glucose	Haupka et al., 2020
C	*E. coli*	Whole-cell biotransformation	Overexpression of RaiP from *S. japonicus* and addition of 4% ethanol and 10 mM H_2_O_2_	29.12	0.44	L-Lysine HCl	Cheng et al., 2018b
D	*E. coli*	Whole-cell biotransformation	Combination of native RaiP, KivD, PadA, KatE, and LysP, without addition of ethanol and H_2_O_2_	52.24	0.38	L-Lysine HCl	This study

*5AVA, 5-Aminovalerate; DavB, Lysine 2-monooxygenase; DavA, δ-Aminovaleramidase; RaiP, Lysine α-oxidase; LdcC, Lysine decarboxylase; PatA, Putrescine transaminase; PatD, γ-Aminobutyraldehyde dehydrogenase; PP2911, 4-Aminobutyrate; LysP, Lysine permease; Puo, Monooxygenase putrescine oxidase; KivD, Ketoacid decarboxylase; KatE, Catalase; PadA, Aldehyde dehydrogenase.*

The promiscuous α-ketoacid decarboxylase (KivD) has been demonstrated in the decarboxylation of α-ketoacids (Atsumi et al., 2008; Chen et al., 2017). In its native pathway, KivD catalyzes a wide variety of α-ketoacids into aldehydes (Xiong et al., 2012; Jambunathan and Zhang, 2014; Wang et al., 2017). Compared with the substrates of wild-type KivD, are mainly smaller substrates, such as 2-ketoisovalerate and α-ketoadipate (Zhang et al., 2008; Wang et al., 2017), KivD mutants are relatively longer, such as 2-keto-4-methylhexanoate and 2-keto-3-methylvalerate (Zhang et al., 2008). Overexpression of KivD from *Lactococcus lactis* (*L. lactis*) and alcohol dehydrogenase 2 (ADH2) in *Escherichia coli*, 1-propanol could be successfully produced from 2-ketobutyrate with a final titer of 2 g/L (Shen and Liao, 2008).

In this study, 5AVA was synthesized using 2-keto-6-aminocaproate as intermediate, which is related to the involvement of three key enzymes—RaiP, KivD, and aldehyde dehydrogenase (PadA)—as seen in Figure 1D. Compared with the wild type, the two mutants of KivD in residues F381 and M461 showed higher substrate recognition and catalytic efficiency. Moreover, the overexpression of KatE and LysP, contributes to the removal of H_2_O_2_ and the transport of L-lys, thereby increasing the production of 5AVA, respectively. As can be expected, this artificial pathway has a potential prospect in industrial application, which enhances the value of L-lys and produces 5AVA efficiently in engineered *E. coli*.

## Materials and Methods

### Strains and Plasmids

The strains and plasmids involved in this work are listed in Table 2. The nucleotide sequences of genes *raiP* from *S. japonicus*, *kivD* from *L. lactis* and *padA* from *E. coli* are available in the GenBank database with the accession numbers of MG423617 (Cheng et al., 2018a), AIS03677.1 (McCulloch et al., 2014) and NP_415903.4 (Riley et al., 2006), respectively. In order to establish the synthetic pathway, the *raiP*, *padA*, and *kivD* genes were inserted into pET21a, and then the plasmid pET21a-*raiP-padA-kivD* was generated, which was also named as pETaRPK. Primers for saturation mutation of KivD are listed in Supplementary Table 1. *kivD* was replaced by *kivD*^#^ (*kivD* with F381A/V461A mutations) to form the engineered pET21a-*raiP-padA-kivD^#^*, also named as pETaRPK^#^. The lysine permease gene *lysP* from *E. coli* (GenBank accession No. WP_000253273.1) was amplified from plasmid pLMAIP-04 (Cheng et al., 2018a), and the catalase gene *katE* (GenBank accession No. AAT48137.1) from *E. coli* MG1655. In order to remove H_2_O_2_, accelerate transportation of L-lys and reduce energy consumption, the *katE*, and *lysP* genes were firstly constructed in another single operon with the transcriptional order of *katE-lysP*, and then the engineered pZA22-*katE-lysP* was produced, also named as pZAKL. In addition, *E. coli* BL21 (DE3) with knocked out *cadA* was transformed with the plasmid pCJ01, pETaRPK, pETaRPK^#^, pETakatE, or pETaKL to obtain the strains CJ02, CJ06, CJ07, CJ08, or CJ09, respectively.

**TABLE 2:** Strains and plasmids used in this study.

Strains or plasmids	Description	Sources
**Strains**		
DH5α	Wild type	Novagen
BL21(DE3)	Wild type	Novagen
ML03	*E. coli* BL21(DE3) *ΔcadA*	Cheng et al., 2018a
CJ00	*E. coli* BL21(DE3) harboring plasmid pET21a	Cheng et al., 2018b
CJ01	*E. coli* BL21(DE3) harboring plasmid pCJ01	Cheng et al., 2018b
CJ02	*E. coli* ML03 harboring plasmid pCJ01	Cheng et al., 2018b
CJ05	*E. coli* BL21(DE3) harboring plasmid pETaRPK	This study
CJ06	*E. coli* ML03 harboring plasmid pETaRPK	This study
CJ07	*E. coli* ML03 harboring plasmid pETaRPK^#^	This study
CJ08	*E. coli* ML03 harboring plasmid pETaRPK^#^ and pZAkatE	This study
CJ09	*E. coli* ML03 harboring plasmid pETaRPK^#^ and pZAKL	This study
**Plasmids**		
pZA22	Empty plasmid used as control, Kan^*R*^	Cheng et al., 2019
pCJ01	pET21a-*raiP*, pET21a carries a L-Lysine α-oxidase gene (*raiP*) from *S. japonicus* with *Nde*I and *Bam*HI restrictions, Amp^*R*^	Cheng et al., 2018b
pETaRPK	pET21a-*raiP-kivD-padA*, pET21a carries a L-Lysine α-oxidase gene (*raiP*) from *S. japonicus*, a α-ketoacid decarboxylase gene (*kivD*) from *L. lactis* and a aldehyde dehydrogenase gene (*padA*) from *E. coli*, Amp^*R*^	This study
pETaRPK^#^	pET21a-*raiP-kivD*^#^-*padA*, pET21a carries a L-Lysine α-oxidase gene (*raiP*) from *S. japonicus*, a α-ketoacid decarboxylase mutant (F381A/V461A) gene from *L. lactis* and a aldehyde dehydrogenase gene (*padA*) from *E. coli*, Amp^*R*^	This study
pZAkatE	pZA22-*katE*, pZA22 carries a catalase gene (*katE*) from *E. coli*, Kan^*R*^	This study
pZAKL	pZA22-*katE-lysP*, pZA22 carries a catalase gene (*katE*) from *E. coli* and a lysine permease gene (*lysP*) from *E. coli*, Kan^*R*^	This study

### Cultivation Medium and Conditions

The *E. coli* strains harboring the corresponding plasmids were streaked onto Luria-Bertani (LB) agar plates with appropriate antibiotics at 37°C for overnight. Engineering strains used for shake flask fermentation were cultured in the medium containing 5 g/L yeast extract, 10 g/L tryptone, 15 g/L glucose, 0.1 g/L FeCl_3_, 2.1 g/L citric acid⋅H_2_O, 2.5 g/L (NH_4_)_2_SO_4_, 0.5 g/L K_2_PO_4_⋅3H_2_O, 1.0 mM MgSO_4_, 3 g/L KH_2_PO_4_, and 0.5 mM thiamine diphosphate (ThDP) with appropriate antibiotics. After the OD_600_ of the strains reached 0.5, 0.5 mM of isopropyl β-D-thiogalactoside (IPTG) and 6.5 g/L of L-lys HCl were added.

Fed-batch biotransformation of engineering strains were conducted in a 5.0 L fermenter. The composition of the medium was described in our previous report as follows: glucose, 55 g/L; MgSO_4_⋅7H_2_O, 1.6 g/L; FeSO_4_⋅7H_2_O, 0.00756 g/L; (NH_4_)_2_SO_4_, 1.6 g/L; citric acid, 2 g/L; K_2_HPO_4_⋅3H_2_O, 7.5 g/L; Na_2_SO_4_, 0.02 g/L; ZnSO_4_, 0.0064 g/L; Cu_2_SO_4_⋅5H_2_O, 0.0006 g/L; CoCl_2_⋅6H_2_O, 0.004 g/L (Cheng et al., 2018a). The pH was controlled at 6.7–6.9 by the automatic addition of NH_3_⋅H_2_O, and the temperature was set at 30°C. Antifoam 289 was gradually added to prevent the formation of foam during biotransformation. The initial concentration of L-lys HCl was 40 g/L. The concentration of glucose and L-lys were maintained around 15 and 20 g/L during the whole fermentation process, respectively.

### Protein Expression and Purification

The media for protein expression was supplemented by 0.5 mM ThDP in LB at 37°C. At an OD_600_ of 0.5, 0.5 mM of IPTG was added and then cultured at 20°C for 16 h, cells were washed with potassium phosphate buffer (KPB, 50 mM, pH 8.0) and disrupted by sonication in an ice bath of 50 mM KPB. The enzymes were purified with AKTA Purifier 10 using a Ni-NTA column (Cheng et al., 2019). The concentration of protein was measured by SpectraMax M2^*e*^ at 280 nm. The detections of 5AVA and L-lys were reported in our previous work (Cheng et al., 2018b).

### Enzyme Assay

The oxidation activity of RaiP was measured according to the concentration of hydrogen peroxide (Cheng et al., 2018b). The decarboxylation activity of KivD and KivD mutations (KivD^∗^) were determined at 30°C, using a coupled enzymatic assay (Wang et al., 2017). The reaction mixture contained 1.0 mM NAD^+^, 1.1 μM PadA, 1.1 μM RaiP, 0.85 μM KivD, or KivD^∗^ and different concentrations of L-lys in assay buffer (50 mM KPB, pH 8.0, 1 mM MgSO_4_, 1.0 mM TCEP, 0.5 mM ThDP). The reactions began with the addition of the substrate L-lys, and the formation of NADH was monitored at 340 nm with the extinction coefficient of 6.22 mM^–1^ cm^–1^.

### Homology Modeling, Substrate Docking, and Molecular Dynamic Simulation

The theoretical structure of native KivD and mutant KivD^#^ (KivD with F381A/V461A mutations) (PDB: 2VBF), both were generated by SWISS-MODEL online Server^1^. The 3D structural comparison between KivD and KivD^#^ was revealed using PyMOL 2.2. The ligand 2K6AC was docked into the pocket of KivD or KivD^#^ using AutoDock 4.2.6 package, where the lowest energy conformation in the largest cluster was considered to be the approximately natural complex model (Xie et al., 2019; Tahara et al., 2020). Molecular dynamic (MD) simulation was used to simulate the relationship between structure and function of biomacromolecules in solution in this study (Wu et al., 2020). Two comparative MD simulations at 300 K were executed for KivD and KivD-2K6AC systems with AMBER 18 package (Zuo et al., 2017; Wu et al., 2020).

## Results and Discussion

### Construction of an Artificial Synthetic Route for the Biosynthesis of 5AVA in *E. coli*

**FIGURE 2:**
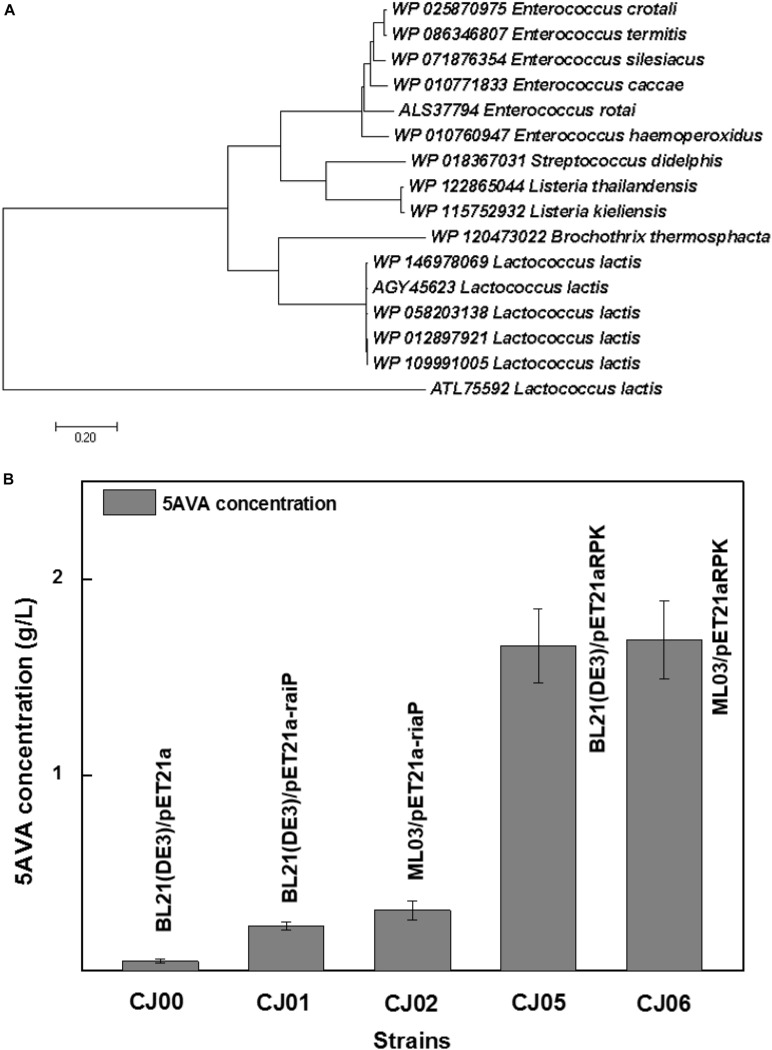
Function identification of α-ketoacid decarboxylase subfamily. **(A)** The phylogenetic relationship of α-ketoacid decarboxylase subfamily. WP 012897921 *Lactococcus lactis* was the α-ketoacid decarboxylase used in this study. All α-ketoacid decarboxylase genes were downloaded from NCBI by blastp against the nr database. The genes were from the species, *L. lactis*, *Enterococcus crotali*, *Enterococcus termitis*, *Enterococcus Silesiacus*, *Enterococcus Caccae*, *Enterococcus rotai*, *Enterococcus haemoperoxidus*, *Streptococcus didelphis*, *Listeria thailandensis*, *Listeria kieliensis*, and *Brochothrix thermosphacta*. The maximum-likelihood tree was constructed by MEGA (Li et al., 2019a, b). **(B)** An artificial pathway confirmed for the biosynthesis of 5AVA. 6.5 g/L of L-Lysine HCl was as substrate. All experiments were performed a minimum of three independent sets. All error bars represent standard deviations with *n* ≥ 3 independent reactions.

Figure 1D showed a heterogeneous artificial route for the bioconversion of L-lys to 5AVA. The designed artificial biosynthetic pathway of 5AVA consists of three steps: (1) deamination of L-lys to form intermediate 2K6AC via RaiP; (2) decarboxylation of 2K6AC to produce 5-aminopentanal via KivD; (3) oxidation of 5-aminopentanal to 5AVA via PadA. Firstly, a plasmid pETaRPK was constructed and introduced into *E. coli* ML03 to obtain the strain CJ05, with the co-expression of RaiP, KivD, and PadA under a T7 promoter. To reduce the degradation of L-lys to cadaverine, the lysine decarboxylase gene *cadA* was knocked out to obtain the strain CJ06. The maximum-likelihood tree was displayed in Figure 2A. Notably, 5AVA could be produced in strains CJ01, CJ02, CJ05, and CJ06. As shown in Figure 2B, the control strain CJ00 only produced 0.06 g/L 5AVA from 6.5 g/L L-lys HCl with the consumption of 0.01 g/g L-lys. For engineered strain CJ01, a titer of 0.23 g/L 5AVA was acquired. Moreover, the strain CJ05 produced 1.66 g/L of 5AVA by this artificial pathway (see Figure 1D), with a yield increase of 774% compared to the single gene pathway (see Figure 1C). These results demonstrate the feasibility of this proposed artificial 5AVA pathway.

### Molecular Docking and MD Simulation of KivD and KivD^#^

**FIGURE 3:**
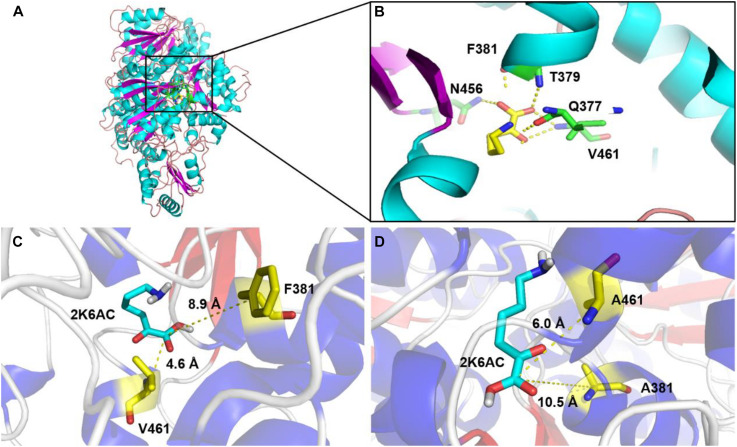
Homology modeling and structure comparison between KivD and KivD^#^(F381A/V461A). **(A)** Overall architecture of the KivD system; **(B)** Interactions of the ligand 2K6AC with their surroundings in KivD system; Binding pocket of *L. lactis* KivD (PDB: 2VBF) **(C)** and KivD^#^(F381A/V461A) **(D)** complexed with its substrate 2K6AC. The active pocket of KivD which is constituted by a number of hydrophobic residues, including F381, T379, and V461. KivD, α-ketoacid decarboxylase.

**FIGURE 4:**
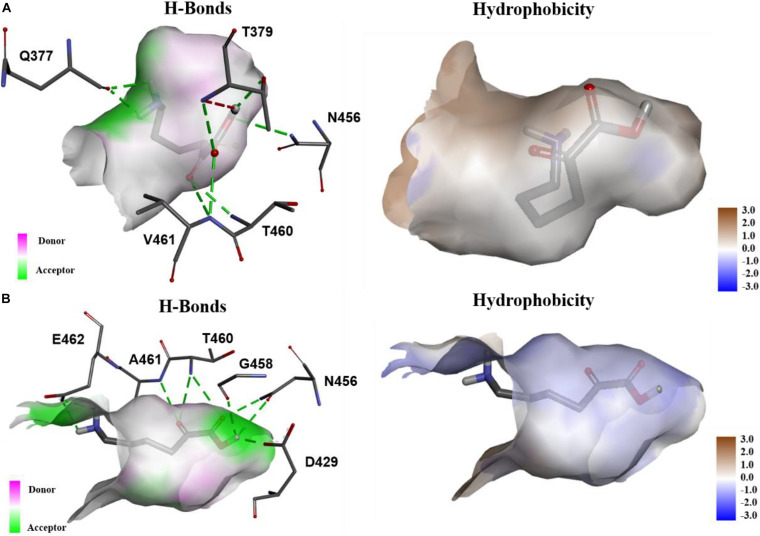
The analysis of hydrogen bonds and hydrophobicity. **(A)** The hydrogen bonds formed of KivD and 2K6AC, and the hydrophobicity of the active pocket in KivD; **(B)** The hydrogen bonds formed of KivD^#^(F381A/V461A) and 2K6AC, and the hydrophobicity of the active pocket in KivD^#^(F381A/V461A).

**FIGURE 5:**
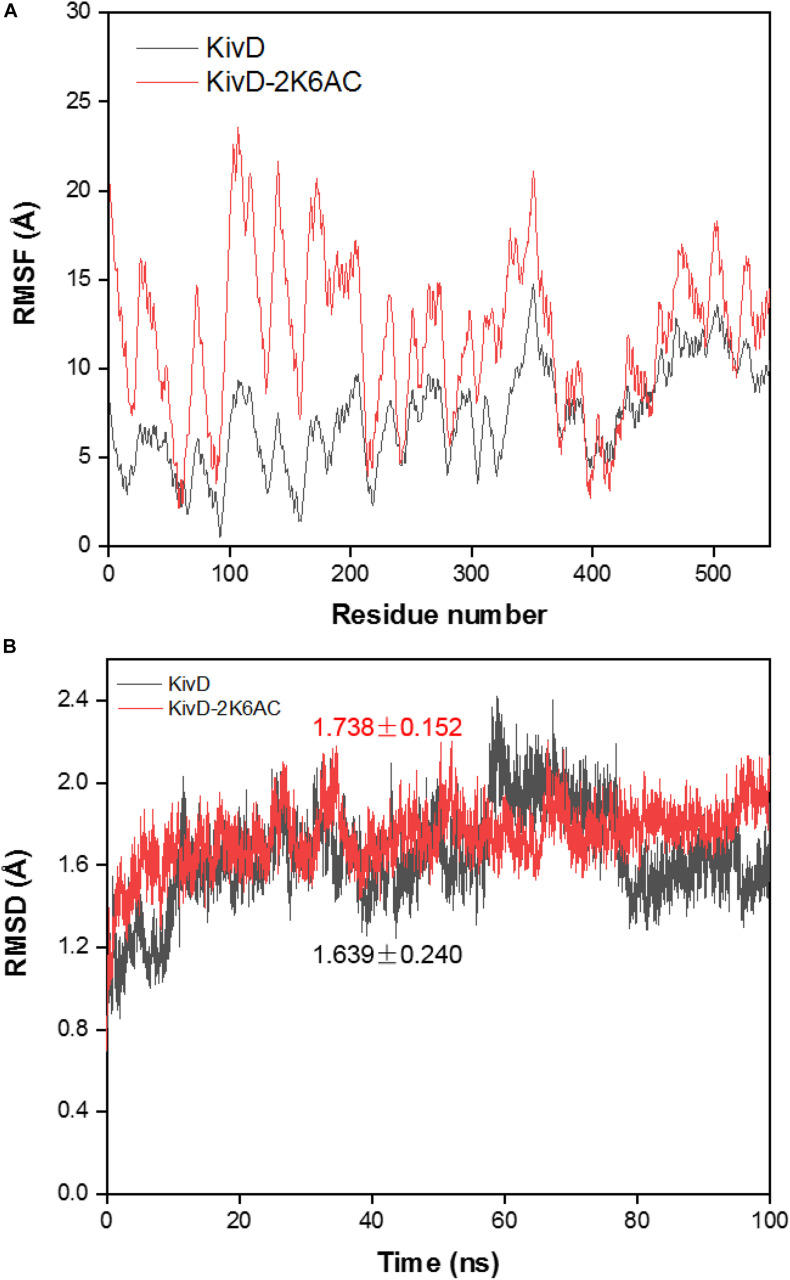
Molecular dynamic simulation of KivD and KivD-2K6AC. **(A)** RMSD of the C_α_ atoms in the KivD and KivD-2K6AC versus simulation time. **(B)** RMSF distribution of the C_α_ atom in the KivD and KivD-2K6AC. RMSD, Root mean squared deviation; RMSF, Root mean squared fluctuation.

In order to explore the mechanism of the 5AVA increase in mutants, molecular docking and MD simulation were discussed (Xiang et al., 2019). The structures of KivD and KivD^#^ both are mainly composed of 23 α-helices and 17 β-strands, containing a large activity pocket. Compared with that of KivD, the structure of KivD^#^ remains almost unchanged. Nevertheless based on homology modeling analysis, the catalytic channel of mutant KivD^#^ was enlarged. According to bioinformatics and crystal structure information (PDB: 2VBF) (Berthold et al., 2007), residues F381 and V461 are the two key residues for KivD catalysis (see Figure 3). Modeling and molecular docking of KivD with ligand 2K6AC further highlight the residues involved in substrate recognition. As shown in Figure 3, the substrate docking results indicated that the distances of ligand 2K6AC with F381A, V461A active sites both became farther. The docking results of KivD and 2K6AC showed that 2K6AC formed eight hydrogen bonds with the side chain Q377, T379, N456, T460, and V461. 2K6AC formed nine hydrogen bonds with the side chain D429, N456, G458, T460, A461, and E462 of KivD^#^ (Figure 4). At the same time, the surface hydrophobicity of the catalytic pocket in mutated protein KivD^#^ has also changed (Figure 4). We speculated that the increase in catalytic activity of KivD^#^ may be due to the expansion of catalytic channel and the formation of more hydrogen bonds, the expansion that is likely to result in a change in the conformation of the small molecule 2K6AC which was beneficial to stretch. Through the MD simulations, the results of the root mean squared deviation (RMSD) showed that the RMSD of the KivD system and the complex system KivD-2K6AC were basically maintained at 1.639 ± 0.240 Å and 1.738 ± 0.152 Å (see Figure 5B), which indicated that the MD simulation process was reliable (Zuo et al., 2017). As seen in Figure 5A, there are four fragments of the KivD with lower root mean squared fluctuation (RMSF) values, that is G58-L69, T212-N223, T379-F388, and D457-H466. These four fragments are located near ThDP, which may be related to the activity of KivD (Zuo et al., 2017; Liu et al., 2019).

KivD mutations (F381A/V461, F381L/V461, F381/V461A, F381/V461L, and F381A/V461A) displayed enhanced activities in Table 3. The KivD F381A/V461A (KivD^#^) showed the greatest activity shown in Table 3. KivD^#^ displays a K_*m*_ value of 2.52 mM, a K_*cat*_ value of 562.16 s^–1^ and a K_*cat*_/K_*m*_ value of 223.08 mM^–1^s^–1^ with 2K6AC used as the substrate shown in Table 3.

**TABLE 3:** Kinetic parameters of α-ketoacid decarboxylase KivD mutants (KivD*) on 2-keto-6-aminocaproate (2K6AC).

Enzyme	V_*max*_ (mM min^–1^)	K_*m*_ (mM)	V_*max*_/K_*m*_ (h^–1^)
KivD (F381/V461)	22.69 ± 3.28	6.67 ± 0.26	204.08
KivD*(F381L/V461)	22.56 ± 3.12	5.45 ± 0.22	248.36
KivD*(F381A/V461)	27.25 ± 2.87	3.75 ± 0.18	436.02
KivD*(F381/V461L)	22.63 ± 2.48	6.10 ± 0.23	222.55
KivD*(F381/V461A)	25.88 ± 3.00	3.99 ± 0.15	389.24
KivD*(F381A/V461A)	28.67 ± 3.69	2.52 ± 0.11	682.64

*Data are presented as means ± STDV calculated from three replicate biotransformation experiments. The KivD^∗^ activity toward 2K6AC was performed on 50 mM KPB (pH 8.0), 1 mM MgSO_4_, 1.0 mM TCEP, 0.5 mM ThDP, 1.0 mM NAD^+^, 1.1 μM PadA, 1.1 μM RaiP, 0.85 μM KivD, or KivD^∗^ and different concentrations of L-lys.*

### Overexpression of Catalase KcatE and Lysine Permease LysP Favoring the Increase of 5AVA Production

There are four strategies used in this study to increase the production of 5AVA. Firstly, lysine decarboxylase gene *cadA* was knocked out and L-lys HCl was selected as the industrial substrate for enhancing the utilization of L-lys (Cheng et al., 2018a, b, 2020). Thirdly, H_2_O_2_ could inhibit cell growth, thus affecting the production of goal production (Niu et al., 2014). In Liu’s experiments, through the expression of catalase, the content of H_2_O_2_ was significantly reduced, and the output of α-ketoglutarate was greatly increased (Liu et al., 2017). In this study, the co-expression of *katE*, *raiP*, *kivD*^#^, and *padA* in strain CJ08 yielded 1.88 g/L of 5AVA without addition of catalase, there was no significant difference compared to strain CJ07 (Table 4). In fact, the H_2_O_2_ generated by RaiP in this work was instantly eliminated by KatE. The data in rows 5 and 7 of Table 4 showed that the overexpression of *katE* did not significantly increase the OD_600_ and the production of 5AVA during shake flask fermentation. On the contrary, it decreased the OD_600_, possibly because the increase in gene expression caused an increase in cell burden (Camara et al., 2017). However, in the fermentation tank, H_2_O_2_ could significantly inhibit cell growth, resulting in limited production of 5AVA (Cheng et al., 2018b, 2020). In addition, a lysine transporter gene *lysP* was overexpressed and inserted into the plasmid pZAkatE to form a new plasmid pZAKL. As shown in Table 4, strain CJ09 produced 1.93 g/L of 5AVA.

**TABLE 4:** 5AVA synthesis by engineered strains in 250 mL flasks.

Strains	Time (h)	Cell density (OD_600_)	Glucose consumed (g/L)	5AVA production (g/L)	Statistic analysis^*a*^	5AVA yield (g/g)^*b*^
CJ06	12	5.24 ± 0.38	7.22 ± 0.33	0.85 ± 0.04	–	0.19 ± 0.03
	24	8.15 ± 0.52	11.36 ± 0.46	1.69 ± 0.03	–	0.35 ± 0.03
CJ07	12	5.19 ± 0.41	7.09 ± 0.25	0.96 ± 0.02	*	0.25 ± 0.01
	24	8.08 ± 0.55	11.25 ± 0.48	1.85 ± 0.02	*	0.39 ± 0.03
CJ08	12	5.14 ± 0.36	7.02 ± 0.28	0.94 ± 0.01	ns	0.25 ± 0.02
	24	7.91 ± 0.46	11.17 ± 0.41	1.88 ± 0.02	ns	0.40 ± 0.03
CJ09	12	5.08 ± 0.33	6.88 ± 0.18	1.01 ± 0.03	*	0.23 ± 0.01
	24	7.85 ± 0.42	11.11 ± 0.39	1.93 ± 0.01	*	0.41 ± 0.02

*Data are presented as means ± STDV calculated from three replicate biotransformation experiments. Statistics were performed by the two-tailed student t-test. ^∗^P < 0.05; ns, not significant. ^a^Statistic analysis of the 5AVA production were performed with every two separated lines. ^b^The yield of 5AVA was calculated based on L-lys consumption. 6.5 g/L L-lys HCl, 15 g/L glucose, 0.5 mM IPTG, 1.0 mM MgSO_4_ and 0.5 mM ThDP were added.*

### Fed-Batch Biotransformation for 5AVA Production

**FIGURE 6:**
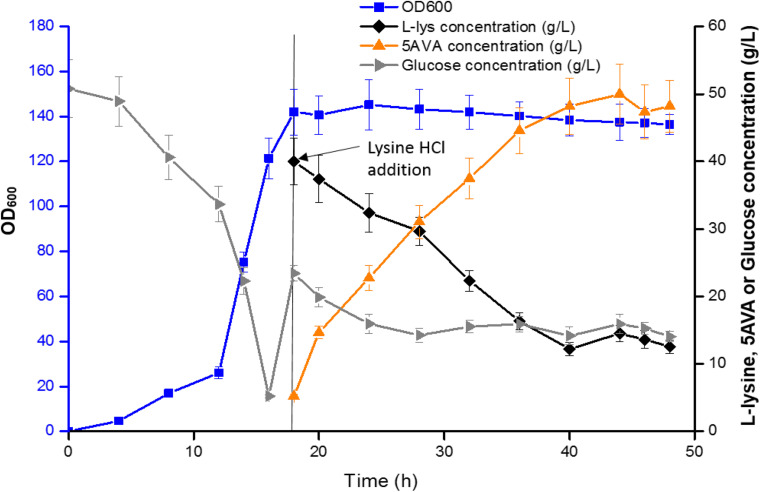
5AVA synthesis by engineered strain CJ09 in a 5 L fermenter. Values and error bars represent the mean and the standard deviation of triplicate cultivations.

Figure 6 showed the results of the fed-batch biotransformation in *E. coli* strain CJ09. Recombinant *E. coli* strain CJ09 grew quickly throughout the biotransformation, reaching the highest cell concentration of an OD_600_ of 142 in 18 h. After the addition of L-lys HCl, 5AVA was accumulated to 48.3 g/L between 18 and 36 h. With the fermentation time increasing to 48 h, 52.24 g/L of 5AVA was successfully acquired. The productivity and yield of 5AVA were 1.09 g/L/h and 0.65 g/g L-lys, respectively. The control strain CJ02 just produced 9.16 g/L 5AVA with a yield of 0.11 g/g L-lys. Interestingly, the expression of KatE in strain CJ08 had no effect on the production of 5AVA in shake flask (Table 4), but it could significantly improve the production of 5AVA to 45.92 g/L in fermentation tank compared to strain CJ07 with a titer of 16.48 g/L. This is because H_2_O_2_ can significantly inhibit the growth of strain CJ07, resulting in OD_600_ of only 40. The above results advocated that the synthetic route developed in this work can effectively produce 5AVA.

Compared with a previous whole cell transformation, the titer of 5AVA based on this synthesis pathway increased by about 79.4% from 29.12 to 52.24 g/L as seen in Table 1; the inhibition of cell growth and enzyme activity by H_2_O_2_ both resulted in the lower yields of 5AVA (Cheng et al., 2018b). Compared with another new synthesis pathway for the fermentative production of 5AVA, in which the titer was only 5.1 g/L (seen in Table 1; Jorge et al., 2017), and the titer was greatly increased in this study. Compared with another whole-cell catalysis work, this synthetic pathway increased the titer of 5AVA by about 3.20% from 50.62 to 52.24 g/L (Cheng et al., 2020). Importantly, the industrial production of 5AVA without the addition of ethanol and H_2_O_2_ was more safe and economical in this study. In terms of reaction mechanism, the new 5AVA synthesis strategy proposed in this work mainly includes three steps: (1) the accumulation of intermediate 6A2KCA by RaiP; (2) the decarboxylation of 6A2KCA to 5-aminopentanal by KivD; (3) the oxidization of 5-aminopentanal to 5AVA by PadA.

## Conclusion

From renewable feedstocks, an artificial pathway in *E. coli* was proposed and optimized to produce 5AVA in this study. Since the inhibition of enzyme activity and cell growth by H_2_O_2_ is the main limiting factor in the production of 5AVA, catalase KatE was overexpressed to decompose H_2_O_2_ to achieve high yield of 5AVA. Finally, an engineered strain CJ09 with RaiP, KivD, PadA, KatE, and LysP overexpression successfully produced 5AVA from biobased L-lys HCl at a final titer of 52.24 g/L. The renewable substrate and simple culture conditions were adopted in this work, while possessing higher yield and less environmental pollution. The improvement of substrate utilization and H_2_O_2_ decomposition efficiency contributes to the increase in the yield of 5AVA, which has the potential to become a common strategy for the sustainable production of other chemicals.

## Data Availability Statement

The original contributions presented in the study are included in the article/Supplementary Material, further inquiries can be directed to the corresponding authors.

## Author Contributions

JC and WT performed the experiments, analyzed the data, and drafted the manuscript. ZL, QL, and XG analyzed the data. JC and DW conceived and coordinated the study. JC, DW, and JZ finalized the manuscript. All authors contributed to the article and approved the submitted version.

## Conflict of Interest

The authors declare that the research was conducted in the absence of any commercial or financial relationships that could be construed as a potential conflict of interest.

## Footnotes

1 https://swissmodel.expasy.org/
