# Supplementary material for: A High-Efficiency Artificial Synthetic Pathway for 5-Aminovalerate Production From Biobased L-Lysine in Escherichia coli
Source: Front Bioeng Biotechnol. 2021 Feb 9;9:633028. doi: 10.3389/fbioe.2021.633028 (PMC7900509; doi:10.3389/fbioe.2021.633028)
Supplement: Supplementary file 1 [file Data_Sheet_1.DOCX]

# Supporting information for:

**A high-efficiency artificial synthetic pathway for 5-aminovalerate production from biobased L-lysine in *Escherichia coli***

**Jie Cheng****^1,^*, Wenying Tu^1^, Zhou Luo^1^, Xinghua Gou^1^, Qiang Li^1^, Dan Wang^2,^*, Jingwen Zhou^3,^***

^1^ *Key Laboratory of Meat Processing of Sichuan Province, Key Laboratory of Coarse Cereal Processing, Ministry of Agriculture and Rural Affairs, College of Food and Biological Engineering, Chengdu University, Chengdu 610106, P. R. China*

^2^ *Department of Chemical Engineering, School of Chemistry and Chemical Engineering, Chongqing University, Chongqing, 401331, P. R. China*

^3^ *National Engineering Laboratory for Cereal Fermentation Technology, Jiangnan University, 1800 Lihu Road, Wuxi, Jiangsu 214122, P. R. China*

^*^ Address Correspondence: Key Laboratory of Meat Processing of Sichuan Province, College of Food and Biological Engineering, Chengdu University, Chengdu 610106, P. R. China

***E-mail*:** [jcheng@cqu.edu.cn](mailto:jcheng@cqu.edu.cn) (J. Cheng); [dwang@cqu.edu.cn](mailto:dwang@cqu.edu.cn) (D. Wang); [zhoujw1982@jiangnan.edu.cn](mailto:zhoujw1982@jiangnan.edu.cn) (J. Zhou)

**Supplementary Table S1**

**List of primers for saturation mutation of KivD used in this study.**

| Primers names | Nucleotide sequence (5’-3’) |
| --- | --- |
| F381A-F | GCTTTCGGTGCAAGCAGCATCTTC |
| F381C-F | TGCTTCGGTGCAAGCAGCATCTTC |
| F381D-F | GACTTCGGTGCAAGCAGCATCTTC |
| F381E-F | GAGTTCGGTGCAAGCAGCATCTTC |
| F381G-F | GGATTCGGTGCAAGCAGCATCTTC |
| F381H-F | CATTTCGGTGCAAGCAGCATCTTC |
| F381I-F | ATCTTCGGTGCAAGCAGCATCTTC |
| F381K-F | AAGTTCGGTGCAAGCAGCATCTTC |
| F381L-F | CTGTTCGGTGCAAGCAGCATCTTC |
| F381M-F | ATGTTCGGTGCAAGCAGCATCTTC |
| F381N-F | AACTTCGGTGCAAGCAGCATCTTC |
| F381P-F | CCTTTCGGTGCAAGCAGCATCTTC |
| F381Q-F | CAGTTCGGTGCAAGCAGCATCTTC |
| F381R-F | CGTTTCGGTGCAAGCAGCATCTTC |
| F381S-F | AGTTTCGGTGCAAGCAGCATCTTC |
| F381T-F | ACGTTCGGTGCAAGCAGCATCTTC |
| F381V-F | GTCTTCGGTGCAAGCAGCATCTTC |
| F381W-F | TGGTTCGGTGCAAGCAGCATCTTC |
| F381Y-F | TACTTCGGTGCAAGCAGCATCTTC |
| F381-R | GCTGGTGCCCTGCTCTGCCACGATG |
| V461A-F | GCTGAACGTGAGATTCACGGCCCGAAC |
| V461C-F | TGCGAACGTGAGATTCACGGCCCGAAC |
| V461D-F | GACGAACGTGAGATTCACGGCCCGAAC |
| V461E-F | GAGGAACGTGAGATTCACGGCCCGAAC |
| V461F-F | TTCGAACGTGAGATTCACGGCCCGAAC |
| V461G-F | GGCGAACGTGAGATTCACGGCCCGAAC |
| V461H-F | CATGAACGTGAGATTCACGGCCCGAAC |
| V461I-F | ATCGAACGTGAGATTCACGGCCCGAAC |
| V461K-F | AAGGAACGTGAGATTCACGGCCCGAAC |
| V461L-F | CTGGAACGTGAGATTCACGGCCCGAAC |
| V461M-F | ATGGAACGTGAGATTCACGGCCCGAAC |
| V461N-F | AACGAACGTGAGATTCACGGCCCGAAC |
| V461P-F | CCTGAACGTGAGATTCACGGCCCGAAC |
| V461Q-F | CAGGAACGTGAGATTCACGGCCCGAAC |
| V461R-F | CGTGAACGTGAGATTCACGGCCCGAAC |
| V461S-F | AGTGAACGTGAGATTCACGGCCCGAAC |
| V461T-F | ACGGAACGTGAGATTCACGGCCCGAAC |
| V461W-F | TGGGAACGTGAGATTCACGGCCCGAAC |
| V461Y-F | TACGAACGTGAGATTCACGGCCCGAAC |
| V461-R | GGTCTAGCCGTCGTTGTTGATG |

**Supplementary Table S2**

**Plasmids for saturation mutation of KivD used in this study.**

|  | Relevant genotype or description | Sources |
| --- | --- | --- |
| pETaRPK*( F381A/V461) | pET21a carries a L-lysine α-oxidase gene (*raiP*) from *S. japonicus*, a α-ketoacid decarboxylase mutant (F381A/V461) gene from *L. lactis* and a aldehyde dehydrogenase gene (*padA*) from *E. coli*, Amp^R^ | This study |
| pETaRPK*( F381C/V461) | pET21a carries a L-lysine α-oxidase gene (*raiP*) from *S. japonicus*, a α-ketoacid decarboxylase mutant (F381C/V461) gene from *L. lactis* and a aldehyde dehydrogenase gene (*padA*) from *E. coli*, Amp^R^ | This study |
| pETaRPK*( F381D/V461) | pET21a carries a L-lysine α-oxidase gene (*raiP*) from *S. japonicus*, a α-ketoacid decarboxylase mutant (F381C/V461) gene from *L. lactis* and a aldehyde dehydrogenase gene (*padA*) from *E. coli*, Amp^R^ | This study |
| pETaRPK*( F381E/V461) | pET21a carries a L-lysine α-oxidase gene (*raiP*) from *S. japonicus*, a α-ketoacid decarboxylase mutant (F381E/V461) gene from *L. lactis* and a aldehyde dehydrogenase gene (*padA*) from *E. coli*, Amp^R^ | This study |
| pETaRPK*( F381G/V461) | pET21a carries a L-lysine α-oxidase gene (*raiP*) from *S. japonicus*, a α-ketoacid decarboxylase mutant (F381G/V461) gene from *L. lactis* and a aldehyde dehydrogenase gene (*padA*) from *E. coli*, Amp^R^ | This study |
| pETaRPK*( F381H/V461) | pET21a carries a L-lysine α-oxidase gene (*raiP*) from *S. japonicus*, a α-ketoacid decarboxylase mutant (F381H/V461) gene from *L. lactis* and a aldehyde dehydrogenase gene (*padA*) from *E. coli*, Amp^R^ | This study |
| pETaRPK*( F381I/V461) | pET21a carries a L-lysine α-oxidase gene (*raiP*) from *S. japonicus*, a α-ketoacid decarboxylase mutant (F381I/V461) gene from *L. lactis* and a aldehyde dehydrogenase gene (*padA*) from *E. coli*, Amp^R^ | This study |
| pETaRPK*( F381K/V461) | pET21a carries a L-lysine α-oxidase gene (*raiP*) from *S. japonicus*, a α-ketoacid decarboxylase mutant (F381K/V461) gene from *L. lactis* and a aldehyde dehydrogenase gene (*padA*) from *E. coli*, Amp^R^ | This study |
| pETaRPK*( F381L/V461) | pET21a carries a L-lysine α-oxidase gene (*raiP*) from *S. japonicus*, a α-ketoacid decarboxylase mutant (F381L/V461) gene from *L. lactis* and a aldehyde dehydrogenase gene (*padA*) from *E. coli*, Amp^R^ | This study |
| pETaRPK*( F381M/V461) | pET21a carries a L-lysine α-oxidase gene (*raiP*) from *S. japonicus*, a α-ketoacid decarboxylase mutant (F381M/V461) gene from *L. lactis* and a aldehyde dehydrogenase gene (*padA*) from *E. coli*, Amp^R^ | This study |
| pETaRPK*( F381N/V461) | pET21a carries a L-lysine α-oxidase gene (*raiP*) from *S. japonicus*, a α-ketoacid decarboxylase mutant (F381N/V461) gene from *L. lactis* and a aldehyde dehydrogenase gene (*padA*) from *E. coli*, Amp^R^ | This study |
| pETaRPK*( F381P/V461) | pET21a carries a L-lysine α-oxidase gene (*raiP*) from *S. japonicus*, a α-ketoacid decarboxylase mutant (F381P/V461) gene from *L. lactis* and a aldehyde dehydrogenase gene (*padA*) from *E. coli*, Amp^R^ | This study |
| pETaRPK*( F381Q/V461) | pET21a carries a L-lysine α-oxidase gene (*raiP*) from *S. japonicus*, a α-ketoacid decarboxylase mutant (F381Q/V461) gene from *L. lactis* and a aldehyde dehydrogenase gene (*padA*) from *E. coli*, Amp^R^ | This study |
| pETaRPK*( F381R/V461) | pET21a carries a L-lysine α-oxidase gene (*raiP*) from *S. japonicus*, a α-ketoacid decarboxylase mutant (F381R/V461) gene from *L. lactis* and a aldehyde dehydrogenase gene (*padA*) from *E. coli*, Amp^R^ | This study |
| pETaRPK*( F381S/V461) | pET21a carries a L-lysine α-oxidase gene (*raiP*) from *S. japonicus*, a α-ketoacid decarboxylase mutant (F381S/V461) gene from *L. lactis* and a aldehyde dehydrogenase gene (*padA*) from *E. coli*, Amp^R^ | This study |
| pETaRPK*( F381T/V461) | pET21a carries a L-lysine α-oxidase gene (*raiP*) from *S. japonicus*, a α-ketoacid decarboxylase mutant (F381T/V461) gene from *L. lactis* and a aldehyde dehydrogenase gene (*padA*) from *E. coli*, Amp^R^ | This study |
| pETaRPK*( F381V/V461) | pET21a carries a L-lysine α-oxidase gene (*raiP*) from *S. japonicus*, a α-ketoacid decarboxylase mutant (F381V/V461) gene from *L. lactis* and a aldehyde dehydrogenase gene (*padA*) from *E. coli*, Amp^R^ | This study |
| pETaRPK*( F381W/V461) | pET21a carries a L-lysine α-oxidase gene (*raiP*) from *S. japonicus*, a α-ketoacid decarboxylase mutant (F381W/V461) gene from *L. lactis* and a aldehyde dehydrogenase gene (*padA*) from *E. coli*, Amp^R^ | This study |
| pETaRPK*( F381Y/V461) | pET21a carries a L-lysine α-oxidase gene (*raiP*) from *S. japonicus*, a α-ketoacid decarboxylase mutant (F381Y/V461) gene from *L. lactis* and a aldehyde dehydrogenase gene (*padA*) from *E. coli*, Amp^R^ | This study |
| pETaRPK*( F381/V461A) | pET21a carries a L-lysine α-oxidase gene (*raiP*) from *S. japonicus*, a α-ketoacid decarboxylase mutant (F381/V461A) gene from *L. lactis* and a aldehyde dehydrogenase gene (*padA*) from *E. coli*, Amp^R^ | This study |
| pETaRPK*( F381/V461C) | pET21a carries a L-lysine α-oxidase gene (*raiP*) from *S. japonicus*, a α-ketoacid decarboxylase mutant (F381/V461C) gene from *L. lactis* and a aldehyde dehydrogenase gene (*padA*) from *E. coli*, Amp^R^ | This study |
| pETaRPK*( F381/V461D) | pET21a carries a L-lysine α-oxidase gene (*raiP*) from *S. japonicus*, a α-ketoacid decarboxylase mutant (F381/V461D) gene from *L. lactis* and a aldehyde dehydrogenase gene (*padA*) from *E. coli*, Amp^R^ | This study |
| pETaRPK*( F381/V461E) | pET21a carries a L-lysine α-oxidase gene (*raiP*) from *S. japonicus*, a α-ketoacid decarboxylase mutant (F381/V461E) gene from *L. lactis* and a aldehyde dehydrogenase gene (*padA*) from *E. coli*, Amp^R^ | This study |
| pETaRPK*( F381/V461F) | pET21a carries a L-lysine α-oxidase gene (*raiP*) from *S. japonicus*, a α-ketoacid decarboxylase mutant (F381/V461F) gene from *L. lactis* and a aldehyde dehydrogenase gene (*padA*) from *E. coli*, Amp^R^ | This study |
| pETaRPK*( F381/V461G) | pET21a carries a L-lysine α-oxidase gene (*raiP*) from *S. japonicus*, a α-ketoacid decarboxylase mutant (F381/V461G) gene from *L. lactis* and a aldehyde dehydrogenase gene (*padA*) from *E. coli*, Amp^R^ | This study |
| pETaRPK*( F381/V461H) | pET21a carries a L-lysine α-oxidase gene (*raiP*) from *S. japonicus*, a α-ketoacid decarboxylase mutant (F381/V461H) gene from *L. lactis* and a aldehyde dehydrogenase gene (*padA*) from *E. coli*, Amp^R^ | This study |
| pETaRPK*( F381/V461I) | pET21a carries a L-lysine α-oxidase gene (*raiP*) from *S. japonicus*, a α-ketoacid decarboxylase mutant (F381/V461I) gene from *L. lactis* and a aldehyde dehydrogenase gene (*padA*) from *E. coli*, Amp^R^ | This study |
| pETaRPK*( F381/V461K) | pET21a carries a L-lysine α-oxidase gene (*raiP*) from *S. japonicus*, a α-ketoacid decarboxylase mutant (F381/V461K) gene from *L. lactis* and a aldehyde dehydrogenase gene (*padA*) from *E. coli*, Amp^R^ | This study |
| pETaRPK*( F381/V461L) | pET21a carries a L-lysine α-oxidase gene (*raiP*) from *S. japonicus*, a α-ketoacid decarboxylase mutant (F381/V461L) gene from *L. lactis* and a aldehyde dehydrogenase gene (*padA*) from *E. coli*, Amp^R^ | This study |
| pETaRPK*( F381/V461M) | pET21a carries a L-lysine α-oxidase gene (*raiP*) from *S. japonicus*, a α-ketoacid decarboxylase mutant (F381/V461M) gene from *L. lactis* and a aldehyde dehydrogenase gene (*padA*) from *E. coli*, Amp^R^ | This study |
| pETaRPK*( F381/V461N) | pET21a carries a L-lysine α-oxidase gene (*raiP*) from *S. japonicus*, a α-ketoacid decarboxylase mutant (F381/V461N) gene from *L. lactis* and a aldehyde dehydrogenase gene (*padA*) from *E. coli*, Amp^R^ | This study |
| pETaRPK*( F381/V461P) | pET21a carries a L-lysine α-oxidase gene (*raiP*) from *S. japonicus*, a α-ketoacid decarboxylase mutant (F381/V461P) gene from *L. lactis* and a aldehyde dehydrogenase gene (*padA*) from *E. coli*, Amp^R^ | This study |
| pETaRPK*( F381/V461Q) | pET21a carries a L-lysine α-oxidase gene (*raiP*) from *S. japonicus*, a α-ketoacid decarboxylase mutant (F381/V461Q) gene from *L. lactis* and a aldehyde dehydrogenase gene (*padA*) from *E. coli*, Amp^R^ | This study |
| pETaRPK*( F381/V461R) | pET21a carries a L-lysine α-oxidase gene (*raiP*) from *S. japonicus*, a α-ketoacid decarboxylase mutant (F381/V461R) gene from *L. lactis* and a aldehyde dehydrogenase gene (*padA*) from *E. coli*, Amp^R^ | This study |
| pETaRPK*( F381/V461S) | pET21a carries a L-lysine α-oxidase gene (*raiP*) from *S. japonicus*, a α-ketoacid decarboxylase mutant (F381/V461S) gene from *L. lactis* and a aldehyde dehydrogenase gene (*padA*) from *E. coli*, Amp^R^ | This study |
| pETaRPK*( F381/V461T) | pET21a carries a L-lysine α-oxidase gene (*raiP*) from *S. japonicus*, a α-ketoacid decarboxylase mutant (F381/V461T) gene from *L. lactis* and a aldehyde dehydrogenase gene (*padA*) from *E. coli*, Amp^R^ | This study |
| pETaRPK*( F381/V461W) | pET21a carries a L-lysine α-oxidase gene (*raiP*) from *S. japonicus*, a α-ketoacid decarboxylase mutant (F381/V461W) gene from *L. lactis* and a aldehyde dehydrogenase gene (*padA*) from *E. coli*, Amp^R^ | This study |
| pETaRPK*( F381/V461Y) | pET21a carries a L-lysine α-oxidase gene (*raiP*) from *S. japonicus*, a α-ketoacid decarboxylase mutant (F381/V461Y) gene from *L. lactis* and a aldehyde dehydrogenase gene (*padA*) from *E. coli*, Amp^R^ | This study |
| PETaRPK^#^( F381A/V461A) | pET21a carries a L-lysine α-oxidase gene (*raiP*) from *S. japonicus*, a α-ketoacid decarboxylase mutant (F381A/V461A) gene from *L. lactis* and a aldehyde dehydrogenase gene (*padA*) from *E. coli*, Amp^R^ | This study |
